# Phage therapy to treat cystic fibrosis *Burkholderia cepacia* complex lung infections: perspectives and challenges

**DOI:** 10.3389/fmicb.2024.1476041

**Published:** 2024-10-18

**Authors:** Jack S. Canning, Daniel R. Laucirica, Kak-Ming Ling, Mark P. Nicol, Stephen M. Stick, Anthony Kicic

**Affiliations:** ^1^Division of Infection and Immunity, School of Biomedical Sciences, Faculty of Health and Medical Sciences, University of Western Australia, Nedlands, WA, Australia; ^2^Wal-Yan Respiratory Research Centre, The Kids Research Institute Australia, The University of Western Australia, Nedlands, WA, Australia; ^3^School of Population Health, Curtin University, Bentley, WA, Australia; ^4^Division of Infection and Immunity, School of Biomedical Sciences, Marshall Centre, University of Western Australia, Perth, WA, Australia; ^5^Department of Respiratory and Sleep Medicine, Perth Children’s Hospital, Nedlands, WA, Australia; ^6^School of Medicine and Pharmacology, Centre for Cell Therapy and Regenerative Medicine, The University of Western Australia and Harry Perkins Institute of Medical Research, Nedlands, WA, Australia

**Keywords:** bacteriophage, *Burkholderia*, antimicrobial resistance, phage therapy, cystic fibrosis

## Abstract

*Burkholderia cepacia* complex is a cause of serious lung infections in people with cystic fibrosis, exhibiting extremely high levels of antimicrobial resistance. These infections are difficult to treat and are associated with high morbidity and mortality. With a notable lack of new antibiotic classes currently in development, exploring alternative antimicrobial strategies for *Burkholderia cepacia* complex is crucial. One potential alternative seeing renewed interest is the use of bacteriophage (phage) therapy. This review summarises what is currently known about *Burkholderia cepacia* complex in cystic fibrosis, as well as challenges and insights for using phages to treat *Burkholderia cepacia* complex lung infections.

## 1 Introduction: antimicrobial resistance and cystic fibrosis

The incidence of antimicrobial resistance (AMR) continues to increase globally, with current antibiotics insufficient to meet the burden of resistance and little investment into antibiotic discovery pipelines (Murray et al., 2022). Notably, respiratory infections display some of the highest rates of resistance, accounting for the majority of deaths attributable to AMR (Murray et al., 2022). This is a concern for people living with chronic respiratory diseases like cystic fibrosis, who are prone to persistent bacterial lung infections that can develop resistance (Garcia-Clemente et al., 2020). Cystic Fibrosis (*CF*) is an autosomal recessive genetic disorder affecting more than 160,000 people globally (Guo et al., 2022). It is characterised by poor mucociliary clearance leading to persistent infection, inflammation, and airway damage eventually resulting in respiratory failure (Montgomery et al., 2017) and early mortality (Blanchard and Waters, 2022). Early life respiratory infections in *CF* are primarily caused by *Staphylococcus aureus*, with *Pseudomonas aeruginosa* becoming the dominant pathogen in adulthood, chronically infecting up to 70% of individuals (Blanchard and Waters, 2022; Magiorakos et al., 2012). Treatment with repeated courses of antibiotics typically results in *CF* pathogens developing resistance, and presently, AMR prevalence in *CF* is as high as 67% (Cystic Fibrosis Foundation Patient Registry, 2021; Ahern et al., 2022; Bonyadi et al., 2022). The use of *CF* transmembrane conductance regulator (CFTR) modulator therapies has decreased some of the *CF* burden of infection by delaying airway colonisation (Harvey et al., 2022). However, understanding of how modulator therapy impacts chronic infections and bacterial exacerbations is still limited (Saluzzo et al., 2022). CFTR modulators have been shown to be ineffective at eradicating chronic antimicrobial resistant *P. aeruginosa* and other *CF* pathogens, indicating that modulators alone are not suitable for managing infection (Hisert et al., 2017; Mayer-Hamblett et al., 2014). Respiratory infections remain the leading cause of respiratory failure and early mortality for people with *CF* (Blanchard and Waters, 2019), with AMR acquisition further increasing risk of poor health outcomes (Fainardi et al., 2022; John et al., 2021; Durda-Masny et al., 2021). In addition to the substantial impact *P. aeruginosa* and *S. aureus* have on *CF* health outcomes, other bacteria are recognised as causes of less common but highly antimicrobial resistant and fatal infections, one of which is *Burkholderia cepacia* complex (Blanchard and Waters, 2022; Ahern et al., 2022).

## 2 *Burkholderia cepacia* complex

*Burkholderia cepacia* complex (BCC) is a grouping of Gram-negative bacteria comprised of at least 24 biochemically similar but genomically distinct species from the *Burkholderia* genus (Morales-Ruíz et al., 2022). Ubiquitous in the environment but most commonly found in soil (Mahenthiralingam et al., 2005), BCC is a plant pathogen of the allium species, causing onion skin rot (Mahenthiralingam et al., 2005; Jones et al., 2001). In humans, BCC is known as an opportunistic pathogen in individuals with compromised immunity, such as those with chronic granulomatous disease (Holland, 2010). BCC has been found to contaminate hospital liquids, such as nebuliser fluids (Hamill et al., 1995) and mouthwash (Matrician et al., 1998), facilitating nosocomial transmission. BCC is most widely recognised as a respiratory *CF* pathogen. While less common than *P. aeruginosa* and *S. aureus*, BCC is a major clinical concern for people with *CF* of all ages (Cystic Fibrosis Foundation Patient Registry, 2021). Although colonisation with BCC only occurs in up to 4% of individuals with *CF*, approximately 6,500 individuals (Guo et al., 2022), it is associated with accelerated lung function decline (Lambiase et al., 2006; Courtney et al., 2004; Folescu et al., 2015; Downey et al., 2007), increased inflammation (Downey et al., 2007) and higher mortality (Regan and Bhatt, 2019; Jones et al., 2004; Corey and Farewell, 1996) compared to *P. aeruginosa* and *S. aureus* infection. Persistent BCC infections can lead to cepacia syndrome, a life-threatening condition characterised by severe necrotising pneumonia and respiratory failure (Hauser and Orsini, 2015).

The species *B. cenocepacia* and *B. multivorans* account for 70–80% of all BCC infections in *CF* (Jones et al., 2004; Drevinek and Mahenthiralingam, 2010; Boussaud et al., 2008), but the clinical outcomes between these two species may vary. One study reported that patients infected with *B. cenocepacia* had a 18.7% higher 5-year mortality than those infected with *P. aeruginosa* (Jones et al., 2004); however, this was not observed with *B. multivorans* infection. A retrospective study of 247 lung transplant patients with *CF* observed that while BCC infection was generally not associated with an increased incidence of mortality compared to non-BCC infections (Boussaud et al., 2008), the risk of early death was significantly higher in individuals specifically infected with *B. cenocepacia* (Boussaud et al., 2008). These findings are similar to observations in other studies that found individuals with *B. cenocepacia* infection prior to transplantation have significantly decreased 1, 5, and 10-year survival post transplantation compared to uninfected individuals (Stephenson et al., 2015; Alexander et al., 2008; De Soyza et al., 2010). The *B. cenocepacia* ET-12 strain has also been associated with an increased incidence of mortality and lung failure compared to other strains (Somayaji et al., 2020). Therefore, the severe outcomes of BCC infection in *CF* may primarily be attributable to *B. cenocepacia* and its highly virulent epidemic variants. However, despite less reporting on the more uncommon BCC species in *CF*, these can cause infections resulting in equally severe outcomes (Aslam et al., 2019), highlighting all species in the complex as pathogens of clinical concern.

## 3 Current treatment strategies

BCC is intrinsically resistant to several classes of antibiotics, making treatment extremely difficult (Kamal and Dennis, 2015). With a lack of standardised clinical trial data and reporting of eradication strategies for BCC (Regan and Bhatt, 2019; Horsley and Jones, 2012), there remains no consensus on first-line treatment options (Regan and Bhatt, 2019). Clinicians often form treatment plans for chronic infections based on individualised antibiotic susceptibility profiles (Horsley and Jones, 2012; Garcia et al., 2018). Ceftazidime, minocycline and meropenem have been highlighted as potentially effective antimicrobials (Zhou et al., 2007; Aaron et al., 2000), especially when used in combination with other antibiotics (Aaron et al., 2000; Horsley et al., 2011); however, these drugs can result in organ toxicity, precluding long term use (Horsley et al., 2011). This is especially true for colistin, an antibiotic used as a “last-resort” to treat highly resistant gram-negative infections (Alexander et al., 2008; El-Halfawy et al., 2017), that has nephrotoxic and neurotoxic side effects (Bosso et al., 1991). Successful eradication of BCC is not always achievable despite intensive courses of multiple antibiotics (Horsley et al., 2011). In some *CF* care centres, burdensome eradication regimes are sometimes avoided for many BCC-infected individuals, with clinicians citing excessive drug toxicity, lack of efficacy, and high cost, leaving many infected patients with few options for complete eradication (Horsley et al., 2011).

Since the 1980s, lung transplantation has been a well-established and efficacious treatment option for individuals with late stage *CF* lung disease (Pilewski, 2022), improving quality of life and life expectancy (Pilewski, 2022). Infection control is crucial to successful transplantation, as patients often receive intensive courses of immunosuppressant drugs to mitigate the risk of organ rejection (Fishman, 2017). However, clinicians must account for BCC infection before determining eligibility for transplantation, as the outcomes post-transplantation for those infected are significantly worse than non-infected individuals, as previously noted in this review (Stephenson et al., 2015; Alexander et al., 2008; De Soyza et al., 2010). The high risk of mortality associated with BCC preoperative infection has led to the exclusion of infected individuals from transplantation (De Soyza et al., 2010) or, in some cases, complex multidisciplinary management to determine the benefits and risks associated with transplantation (Olland et al., 2011). As a result of its intrinsically high AMR, association with high morbidity and mortality, and status as a barrier to lung transplantation, BCC is notorious in the *CF* community despite a low rate of infection (Jones et al., 2001). These considerations underscore a need to investigate alternatives to treat BCC infections.

## 4 Bacteriophage

One alternative being explored to treat antimicrobial resistant bacterial infections are bacteriophages (phages), which are viruses that infect bacteria (Malik et al., 2017). Phages possess several characteristics that make them ideal candidates for clinical use: (i) they possess high specificity for their target bacteria and minimally impact the human microbiome (Kakasis and Panitsa, 2019), (ii) they are well tolerated by individuals receiving therapy (Aslam et al., 2019; Haidar et al., 2023), (iii) they are naturally occurring and highly abundant in the environment (Kakasis and Panitsa, 2019), and (iv) are efficacious for antimicrobial resistant infections (Kakasis and Panitsa, 2019). These characteristics have increased interest in applying phage therapy for antimicrobial resistant pathogens causing lung infections in *CF*, including BCC.

**Figure 1:**
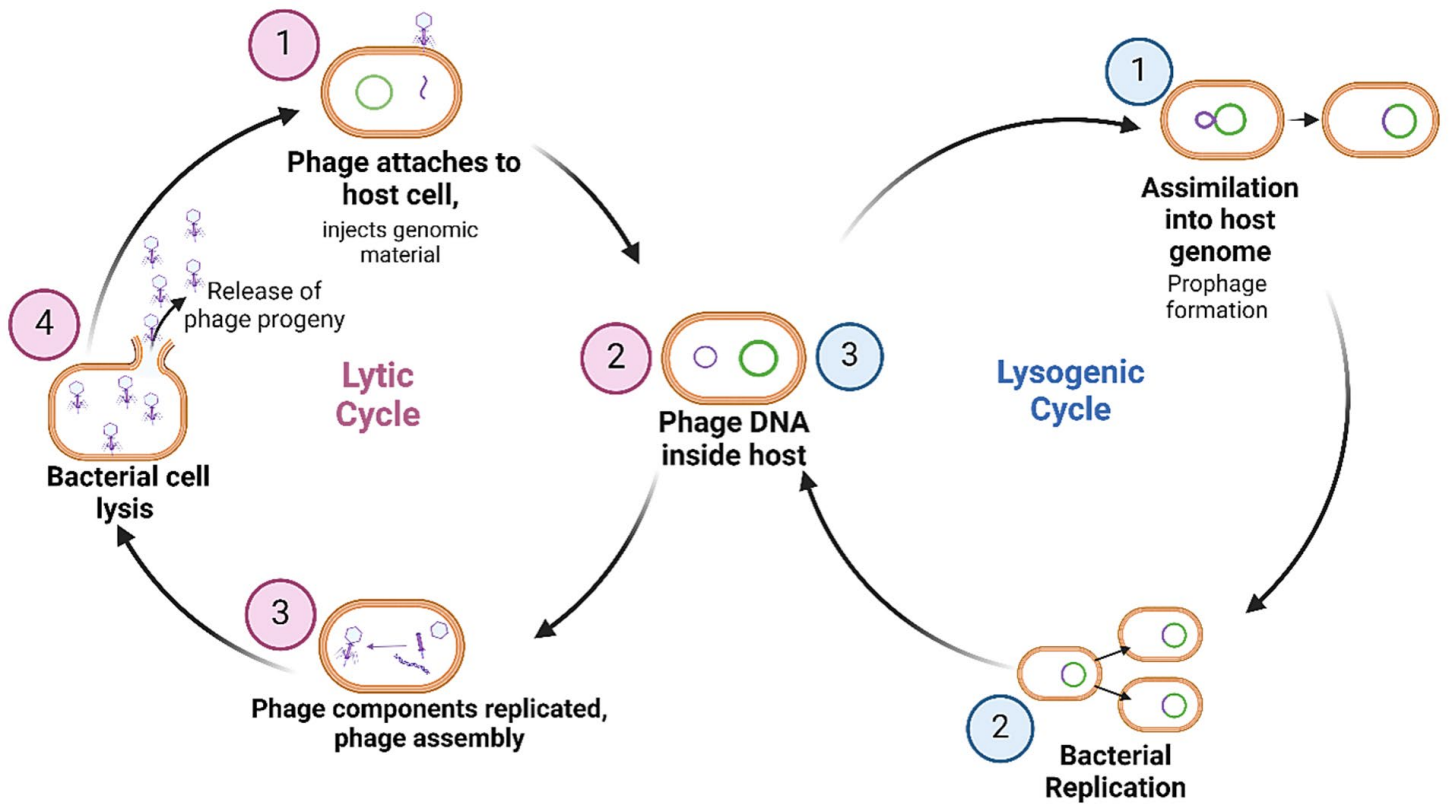
Phage life cycles upon infection of a bacterial host. Currently, phages which are obligately lytic are exclusively preferred for therapy. Figure produced with BioRender.

Phages can broadly follow two main life cycles; the lytic or lysogenic cycles (Kakasis and Panitsa, 2019). In the lytic cycle, phages attach to and infect the bacterial host, utilise the host replication machinery to produce progeny, then induce lysis of the bacterial cell to release new phages and continue the infection cycle (Kakasis and Panitsa, 2019) (Figure 1). Phages that exclusively follow this life cycle are known as obligately lytic (OL) phages. In contrast, the lysogenic cycle results in the integration of the phage genome into the bacterial host genome where it exists as a dormant prophage, replicating whenever the bacteria proliferates (Kakasis and Panitsa, 2019) (Figure 1). Phages that have the ability to enter the lysogenic cycle may also be known as temperate phages, at times alternating life cycles in response to specific triggers (Harrison and Brockhurst, 2017). For use as antimicrobials, OL phages are preferred since the lytic cycle results in bacterial cell death (Kakasis and Panitsa, 2019). Moreover, host genome integration by a temperate phage can result in the horizontal transfer of phage genes which can improve bacterial fitness, enhance virulence and increase bacterial AMR, which would work in opposition to desired therapeutic outcomes (Keen et al., 2017).

## 5 Phages to treat BCC respiratory infections

Phages can be found in any environment in which their target bacterial host is in high abundance, or in microbially dense niches (Latz et al., 2016). Both wastewater (Lynch et al., 2013) and soil (Summer et al., 2006) have been identified as reservoirs of BCC phages. Isolating phages targeting BCC is challenging, as these phages appear less abundant than phages targeting other species (Lauman and Dennis, 2021), and a large proportion are temperate (Semler et al., 2012). Currently, there are five published BCC phages which are confirmed to be OL (Summer et al., 2006; Lynch et al., 2013). Phage JG068, 41,604 bp podovirus, with no lysogeny genes, demonstrated efficacy in rescuing *B. cenocepacia* infected *Galleria mellonella* models (Lynch et al., 2013); however, it has yet to be used in humans. Little is known about the other OL phages, Bcep1, Bcep43, Bcep1A, and Bcep781. None of these phages harbour any known lysogeny genes, and three of the phages, Bcep1, Bcep42, and Bcep781, share significant sequence homology. With few OL phages to study, there remains a lack of clinical research investigating phages as a potential treatment for human BCC respiratory infection. While there have been anecdotal reports of *CF* patients treated with phages targeting BCC (Sacher and Zheng, 2021); there are only two published case reports of compassionate use of BCC phage therapy in individuals with *CF* (Aslam et al., 2019; Haidar et al., 2023) (Table 1).

**Table 1:** A summary of the case information for two published compassionate uses of BCC phage therapy.

Phage	Genome	Genus	Patient	Infection	Time to identification of phage	Treatment course	Immune response to phage therapy	Microbiological response to phage therapy	References
BdPF16phi4281	Not published	Unknown	28-year-old female with *CF*, bilateral lung transplantation Ventilator dependent Ongoing drug related toxicities	*B. dolosa*	5 months after transplant	Phage: 5.3 × 10^6^ – 3.5 × 10^7^ PFU/mL, IV once daily for 2 weeks Twice daily for 4 weeks thereafter Intermittent courses of minocycline, meropenem, and trimethoprim/sulfamethoxazole	No bacteriophage associated adverse events No reporting on phage neutralising antibodies No reporting on bacteriophage detection in sera or respiratory samples Decreased fever and leukocytosis after administration	Week 1: Decreased *B. dolosa* load after phage administration Week 6: Ventilator free for 12 h Week 10: *B.dolosa* sepcticaemia and pneumonia Week 12: PT ceased	Aslam et al. (2019)
Bch7	Not published No genes associated with lysogeny, antimicrobial resistance and virulence	*Bcepfunavirus*	32-year-old female with *CF*, bilateral lung transplantation Ventilator dependent Recurrent *B. multivorans* lung infections for 20 years. *P. aeruginosa, Stenotrophomonas maltophilia*, and *Aspergillus* infections	*B. multivorans*	10 days after referral	3.33 × 10^9^ PFU/mL, AirLife jet nebuliser, 3 times daily for 7 days, One dose administered continuously on day 8 for 3 h Intermittent courses of trimethoprim-sulfamethoxazole, ceftazidime-avibactam, meropenem, minocycline, cefiderocol, levofloxacin, inhaled colistin	No bacteriophage associated adverse events No evidence of phage neutralising antibodies in serum No sera based neutralisation Day 6: Phage DNA detected in circulation Decrease in leukocytosis to within normal limits for 4 days	Day 3–6: Decrease in bacterial load Respiratory aspergillosis Day 5: *B. multivorans* bactarermia Days 6–7: Clinical deterioration *Aspergillus fumigatus* bloodstream infection	Haidar et al. (2023)

In the first case, a patient with *CF* undergoing bilateral transplant became infected with antimicrobial resistant *B. dolosa,* causing pneumonia and septicaemia. Six months after the transplant, the patient was treated with phage BdPF16phi4281, produced by Adaptive Phage Therapeutics (Aslam et al., 2019). No information regarding the phage has been made publicly available, including any sequencing data or *in vitro* bactericidal activity, aside from reporting by the authors that the clinical isolate was susceptible to BdPF16phi4281. The patient remained on broad-spectrum antibiotics during phage therapy, including minocycline, meropenem, and trimethoprim/sulfamethoxazole, which resulted in drug-related toxicity. The patient was also on mechanical ventilation for most of the treatment period. After the initiation of phage therapy, there was a decrease in *B. dosola* load in bronchoalveolar fluid (BALF), and the patient was weaned off mechanical ventilation for a short period. Several clinical metrics improved, including decreased fever and airway secretion, and improved lung function. However, despite these improvements, the patient’s condition eventually worsened, with nephrotoxicity and hepatotoxicity arising from antibiotic treatment, as well as *CF*-related hepatopathy. Both antibiotic and phage therapy were ceased after a planned 12-week course of phage, which resulted in an increased *B. dolosa* load in BALF. The patient eventually succumbed to the infection and its complications; however, there were no reported adverse events associated with the administration of phage therapy over the course of treatment (Aslam et al., 2019). The pharmacokinetics and pharmacodynamics of the administered phage were not assessed; phages were delivered intravenously and the concentration of phages reaching lung tissue was not measured. Moreover, phage susceptibility of *B. dolosa* isolates was not assessed during the course of treatment, meaning potential development of phage resistance could not be detected (Aslam et al., 2019).

In the second case, a patient with a history of *B. multivorans* infections received phage therapy for active infection occurring after a bilateral lung transplant (Haidar et al., 2023). The patient received inhaled nebulised phage therapy with phage Bch7, a myovirus in the *Bcepfunavirus* genus of phages which are not known to contain any lysogeny associated genes, making it a suitable candidate for therapy. Prior to administration of phage, the patient was receiving antibiotics, including trimethoprim/sulfamethoxazole, ceftazidime/avibactam, meropenem, minocycline, cefiderocol, levofloxacin, and inhaled colistin, and was intubated for 29 days prior to therapy. The patient received nebulised phage therapy 3 times daily over 7 days. On day 1, several clinical metrics improved, including reduced need for supplemental oxygen and decreased white blood cell counts. These clinical improvements were short-lived as the patient experienced elevated white blood cell counts and decreased lung function on day 5. Moreover, patient blood samples were positive for *Candida* spp. on day 6, with sputum samples positive for *Aspergillus fumigatus* on days 3 and 6, resulting in the inclusion of antifungals into the treatment regimen. None of these clinical findings correlated with a decrease in *B. multivorans* bacterial load; sputum cultures collected on days 1, 3, 4, 5, and 6 were all positive for *B. multivorans.* The patient also suffered from *B. multivorans* bacteraemia, detected from blood cultures on days 1 and 5. After 7 days of inhaled phage therapy with no significant improvement, a decision was made to administer Bch7 intravenously. Despite additional treatment the patient developed multiple organ failure and acidosis, with no clinical improvement. Sadly the patient died; however, there were no significant adverse events recorded attributable to phage therapy, and blood cultures from day 7 of intravenous phage therapy were negative for *B. multivorans.* Interestingly, phage DNA was detected in tracheal aspirate and BALF samples 6 days post initiation of phage therapy, suggesting a sufficient dose of phages reached the lungs (Haidar et al., 2023).

## 6 Discussion: challenges in the implementation of BCC phage therapy

### 6.1 Current limitations of BCC phages

#### 6.1.1 Lack of available phages and narrow host range

Currently, in most regions worldwide, phages are only available as medicines on the basis of compassionate use, where special approval is given for phage administration to patients who are non-responsive to antibiotic therapy and have exhausted all other treatment options (Patey et al., 2018). Phage therapy is still not widely available for treatment of bacterial infections due to existing barriers and knowledge gaps that must be addressed before full clinical implementation. The lack of phages targeting BCC is the biggest barrier hindering the translation of BCC phage therapy (Semler et al., 2012), slowing research output and precluding clinical use. A keyword search “*Burkholderia*” within Inphared (accessed May 2024), a database of all available phage whole genome sequences uploaded to Genbank, returns 87 results. In contrast, searches with “*Pseudomonas*” and “*Staphylococcus*” return 1,050 and 690 results, respectively.

**Figure 2:**
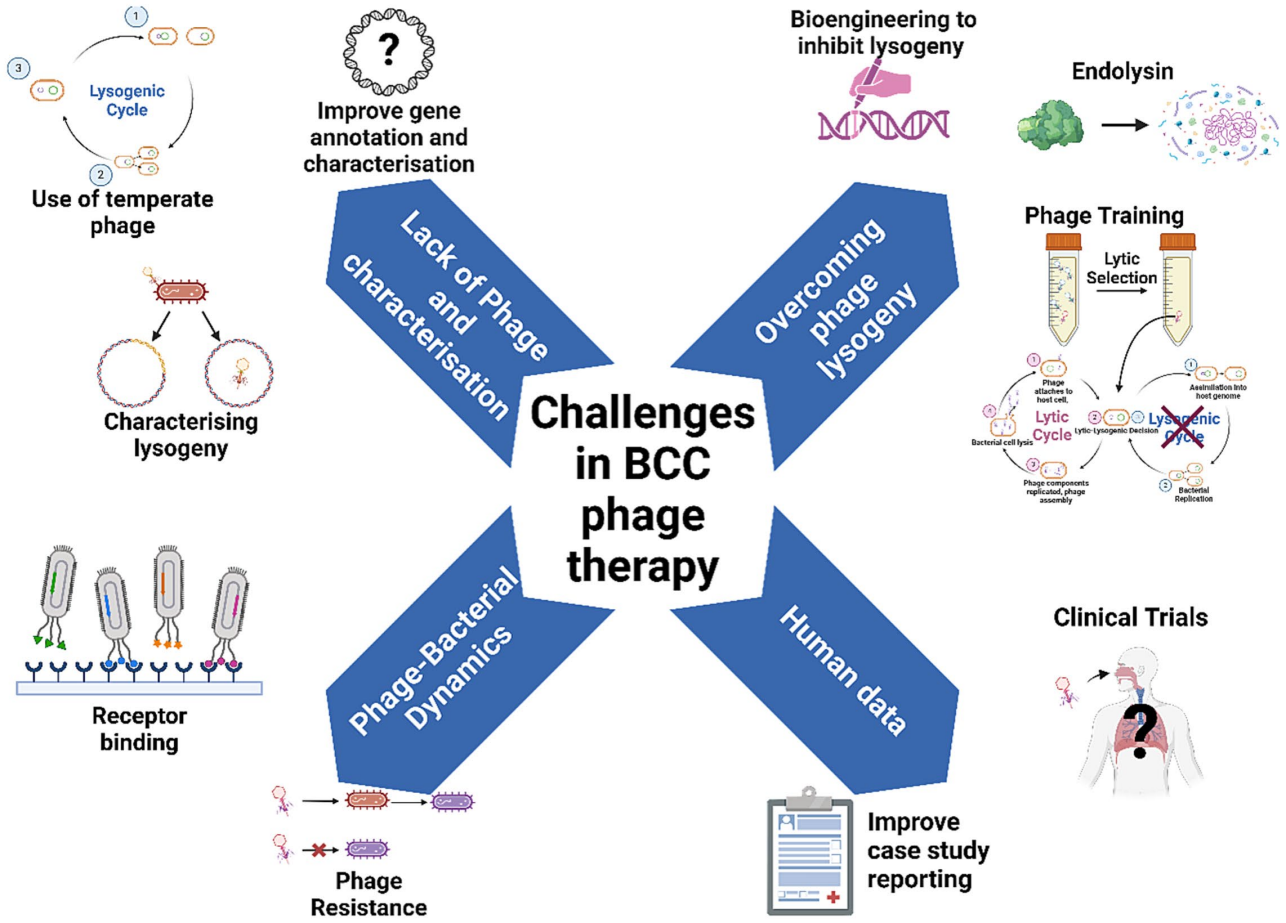
Overcoming challenges to make phage therapy standard practice for BCC lung infections in people with *CF.* Figure produced with BioRender.

Isolating BCC phages has been previously shown to be difficult (Semler et al., 2012), leading researchers to investigate alternative methods of sourcing or harnessing phages for further study. It is not entirely understood why there is such a limited number of BCC phages in the environment; however, it may be due to the fact that BCC phages are known to have relatively narrow host ranges (Seed and Dennis, 2005; Yao et al., 2023), a large proportion of BCC phages are naturally temperate (Lauman and Dennis, 2021), or that the density of BCC in soil microenvironments can vary greatly depending on plant species present (Ramette et al., 2005), resulting in variable reservoirs of BCC phages. One strategy employed to isolate active phages against bacteria which do not have many OL phages is to induce prophage excision from the bacterial host using mitomycin C (Nair et al., 2022). Moreover, attempts to isolate environmental phages targeting BCC have determined that many potential phages were actually excised prophages originating from BCC itself (Lynch et al., 2010; Goudie et al., 2008). These two considerations make BCC phages difficult to study comprehensively and limit the clinical applicability of BCC phages (Figure 2).

#### 6.1.2 Lack of genomic characterisation

Phage lysogeny can result in the acquisition of resistance (Kakasis and Panitsa, 2019) or virulence (Betley and Mekalanos, 1985; Beres et al., 2002) genes by host bacteria. Moreover, the subsequent lysis of bacterial cells possessing phage-encoded virulence factors can result in the release and horizontal transfer of these genes to other bacteria (Keen et al., 2017). Despite this, there is limited evidence to suggest that BCC prophages readily confer virulence factors to their host, and it remains unclear how prophages shape BCC virulence (Roszniowski et al., 2018; Summer et al., 2007). Whole genome sequencing (WGS) has traditionally been used to determine the incidence of these genes within phage populations; however, work on phage genomics is limited because phage gene annotation is still lacking considerably (Ecale Zhou et al., 2019). The current process for phage genome annotation typically involves *de novo* assembly of a phage genome, followed by a comparison of the genome sequence with known sequences to predict the putative function of the encoded proteins. Since these predictions are based on previously sequenced phages, inherent biases in the data can lead to less confident predictions for phages for which limited data is available, including BCC phages (Cook et al., 2021).

Current evidence suggests that a large proportion of the BCC phage population appears to be temperate (Lauman and Dennis, 2021). Moreover, the lack of genomic characterisation of BCC phages means that an even greater proportion of these phages may be capable of lysogeny. For instance, while phages in *Bcepfunavirus* genus do not contain any known genes associated with lysogeny, they are known to contain Par-A/B partition systems, which facilitate plasmid partitioning in bacteria and are associated with phage lysogeny (Štrancar et al., 2023). Therefore, it is entirely possible that phages in the *Bcepfunavirus* genus, including Bch7, are temperate phages. This highlights a gap in knowledge regarding the life cycle that BCC phages can undertake. Characterising genomic composition of BCC phages should be a priority of future studies (Figure 2).

#### 6.1.3 Characterising lysogeny

WGS for the characterisation of BCC phages has its challenges, but can still be used to corroborate findings from functional, lab based assays which may give greater insights into phage activity. BCC phages can be validated as temperate using laboratory assays when this cannot be determined via WGS. Using polymerase chain reaction (PCR) on phage-treated bacteria with phage specific primers, it is also possible to detect phage integration in a target (Lauman and Dennis, 2023). Moreover, it has been shown that integration frequency is based on several factors, including phage host pairing (Lauman and Dennis, 2023). Interestingly, a study assessing lysogeny frequency (LF) in BCC phages found that low LF phages, with an integration frequency of 32% or less, possessed bactericidal activity similar to that of OL (Lauman and Dennis, 2023). In addition, high LF phages were found to act synergistically with low LF and OL phages, suggesting potential for temperate phages to be used in multiple phage (cocktail) formulations (Lauman and Dennis, 2023).

Phage lysogeny can be described on a spectrum and expressed as a frequency, with certain factors influencing this lytic-lysogenic decision. Exposure to DNA-damaging external stress such as antibiotics (Banks et al., 2003), extreme temperatures or pH, community dynamics such as phage and bacterial abundance (Laganenka et al., 2019), host metabolic status (Laganenka et al., 2019) and environmental conditions (Zablocki et al., 2016) all have a role in influencing this lytic-lysogenic switch. A greater understanding on how these factors influence life cycle is needed for safe therapeutic use. For example, a given phage may be capable of bacterial integration within one bacterial host, but not another, and may also integrate in a host at a low enough frequency not to significantly impede bactericidal activity. The collection of empirical, lab-based data regarding BCC phage lysogeny will help further understanding of the factors influencing this decision *in vitro*, and potentially facilitate therapeutic use of temperate phages with lower LF. Until a greater understanding of BCC phage/bacterial dynamics is achieved, particularly regarding phage integration, the suitability of BCC phages should be assessed on a case-by-case basis for treatment (Lauman and Dennis, 2023). While the current research paradigm requires the use of OL phages for therapy, this may not be feasible for BCC given the lack of OL phages isolated. Therefore, for BCC phage therapy, all phages, temperate or not, may need to be investigated for therapeutic use to address this gap (Figure 2). This could help increase the pool of usable phages for critically ill patients infected with BCC in the short term; however, future efforts should also focus on entirely circumventing the possibility of phage lysogeny.

### 6.2 Overcoming Lysogeny

#### 6.2.1 Endolysins

One potential avenue to overcome phage lysogeny could be the isolation and use of phage enzymes known as endolysins (Walmagh et al., 2013) (Figure 2). Endolysins are responsible for the lysis of the bacterial cell at the end of the lytic cycle, and can be individually isolated and expressed using commercially available *Escherichia coli* plasmid expression vectors (Walmagh et al., 2013). Their catalytic activity against bacterial peptidoglycan makes them highly bactericidal in Gram-positive bacteria, but less so for Gram-negative microbes, which are protected by an outer membrane, limiting endolysin penetration into the cell wall (Maciejewska et al., 2017). However, endolysins with activity against Gram-negative bacteria, including BCC, have been isolated and shown to be efficacious (Walmagh et al., 2013; Maciejewska et al., 2017; Lood et al., 2015). Endolysins have been tested against planktonic and biofilm-bound bacteria in animal models of infection, rescuing mice from early mortality compared to untreated controls (Lood et al., 2015; Kim et al., 2020). Current barriers to implementation of endolysins as standalone therapeutics include a lack of clinical research data, standardised manufacturing, and concerns with endolysin stability and method of administration (Khan et al., 2024). The natural endolysin resistance of Gram-negative bacteria may further limit translation of BCC endolysin therapy (Goudie et al., 2008). Interestingly, some endolysins of *P. aeruginosa* phages are lytic against strains of BCC, and vice versa (Maciejewska et al., 2017), suggesting a cross reactive relationship between the endolysins targeting each of these bacterial species. Therefore, *P. aeruginosa* phages could be a potential source of endolysins targeting BCC and merits future investigation.

#### 6.2.2 Phage training

Another method to overcome the temperate life cycle common amongst BCC phages is phage training, which uses random mutation and natural selection to generate desirable phage characteristics (Figure 2). Originally, phage training was applied to increase phage virulence (Morello et al., 2011) and phage host range expansion (Burrowes et al., 2019), which can be achieved using several methods. Evolutionary phage training involves the repeated propagation, collection, and re-propagation of a phage against an ancestral bacterial host, continuously selecting mutants optimised for bactericidal activity against that particular host strain (Ngiam et al., 2024). One study has used evolutionary phage training to increase phage virulence against clinical strains of *P. aeruginosa*, with the trained phage significantly improving survival of infected mice compared to untrained phages (Ngiam et al., 2024). Co-evolution can give insights into bacterial phage resistance and the mutations that drive them, as well as selecting phages to overcome phage resistance (Xu et al., 2023).

Another method of performing phage training, the Applemans protocol, exposes a bacterial host to multiple phages with varying host ranges, to generate recombinant phages with expanded host range. A study using this method expanded the host range of a *P. aeruginosa* phage by combining multiple phages with different host ranges into one cocktail (Burrowes et al., 2019). After 30 propogations of the phage cocktail in each bacterial host strain, a phage with greatly expanded host range, phi176, was created, possessing a host range of each phage combined (Burrowes et al., 2019).

Phage training can also be used to select phages incapable of forming stable lysogens within a population of phages, which may have important therapeutic utility for BCC phages. A study by Chang et al. (2019) successfully isolated an obligately lytic phage variant targeting *S. aureus* by selecting genome deletion variants within a population of phage SA13 using sodium pyrophosphate. The resulting phage, SA13m, possessed a truncated gene within the lysogenic operon, making it unable to form stable lysogens within its host while retaining its lytic activity (Chang et al., 2019). The premise behind this method is that the addition of sodium pyrophosphate, ethylenediaminetetraacetic acid (EDTA), or other chelating agents, causes the cationic bonds which maintain phage structure to break, resulting in spontaneous disintegration of a phage particle (Gutiérrez et al., 2018). Mutant genome deletion variants in a population of phages are more stable in the presence of chelating agents, as the decreased pressure within the capsid head decreases the likelihood of phage disintegration (Gutiérrez et al., 2018). Therefore, small genome variants of a phage population are favourably selected, including those with deletion mutations within lysogeny-associated genes (Gutiérrez et al., 2018). While this method does not specifically select for lysogeny-incapable phage mutants, the methodology could be applied to train BCC phages to be OL over repeated rounds of exposure. Moreover, future phage isolation could be supported by this method, as soil microenvironments have been previously suggested to have the highest proportion of temperate phages (Howard-Varona et al., 2017).

#### 6.2.3 Bioengineering

A more specific method to generate OL mutants of BCC phages is genetic engineering (bioengineering). Bioengineering allows for specific forced mutation or editing of phage genes to enhance or repress characteristics which would make a phage more useful for therapy (Yao et al., 2023). BCC phages have been engineered to increase host range, allowing highly virulent but narrow-acting phages to be more clinically useful for a wide variety of infections (Yao et al., 2023). BCC phage Milgaro, a temperate phage with a high rate of spontaneous mutation generating OL variants, was engineered for enhanced host range via the substitution of its tail fibre with BcetMilo, a “tailocin” or broad-acting tail fibre structure without a capsid head encoded in BCC bacterial genomes (Yao et al., 2023; Yao et al., 2017). This allowed for the generation of a phage mutant which had greatly enhanced therapeutic potential due to its ability to infect a wider variety of BCC clinical isolates (Yao et al., 2023).

There is also potential for bioengineering of phages to circumvent a lysogenic life cycle. The temperate BCC phage KS9 was bioengineered to be OL via the insertion of a disrupted lytic repressor gene in the place of the wild type gene, thus arresting its function (Lynch et al., 2010). Without a functioning lytic repressor gene, the mutant phage, KS9c, was unable to form stable lysogens, effectively preventing phage integration (Lynch et al., 2010). When both KS9 and KS9c phages were tested in a *B. cenocepacia* *Galleria mellonella* larvae infection model, KS9c was able to rescue a slightly greater proportion of larvae from death; however, this difference was not significant. Interestingly, both phages were able to rescue a significant portion of larvae from mortality compared to untreated controls. While the potential for generating bioengineered BCC phages for therapy is an enticing opportunity, the high cost and breadth of expertise required remain major limiting factors in developing such a pipeline (Hussain et al., 2023). Considering the findings of KS9c in *G. mellonella* larvae infection models, it is unclear if bioengineering of phages to repress lysogeny is of any benefit compared to the use of temperate phages, given the disadvantages. While an OL phage is preferred to prevent deleterious gene transfer between phages and bacteria during lysogeny (Keen et al., 2017), this finding reinforces the potential of temperate phages to treat BCC infections where no OL phages are available. Moreover, a greater understanding of BCC phage biology and phage-bacterial interactions is needed to engineer phages with increased efficacy for therapeutic use.

### 6.3 Phage bacterial dynamics

#### 6.3.1 Phage resistance

The potential acquisition of bacterial phage resistance could impact the success or failure of phage therapy and needs to be monitored throughout treatment. Phage resistance is a well-documented issue in phage therapy, particularly for *P. aeruginosa,* where CRISPR-cas and restriction enzyme modification systems can reduce efficacy of therapy (Vaitekenas et al., 2021). Interestingly, BCC is not known to encode CRISPR systems for phage defence (Lauman and Dennis, 2021); however, BCC phage resistance mechanisms are not extensively described. Resistance can be circumvented with careful phage cocktail design and appropriate selection of antibiotics to supplement therapy. Moreover, phage resistance due to receptor mutations in BCC has been found to be associated with fitness trade-offs, including a resensitisation to antibiotics and human sera (Ruest et al., 2023). Previous work has shown that BCC phages often rely on bacterial lipopolysaccharide (LPS) components to bind the host cell and initiate the infection cycle (Lauman and Dennis, 2023; Yao et al., 2017; Stanton et al., 2023; Nzula et al., 2000). However, the inherent genomic variability amongst bacterial complexes means that predicting receptors on the surface of bacteria is difficult. Indeed, several BCC phages have been confirmed as unable to use LPS as a receptor (Lynch et al., 2012). Overall, there is a lack of understanding of the full spectrum of bacterial cell surface components BCC phages can use for binding. Understanding which receptors facilitate the infection cycle could reveal important information on how phage resistance emerges in BCC populations (Figure 2). Studies developing phage/antibiotic cocktails should aim to identify combinations of phages which exert the greatest selection pressures, generate the largest fitness trade-off costs, and suppress the emergence of phage resistance (Vaitekenas et al., 2021) (Figure 2).

### 6.4 Use of BCC phages in humans

#### 6.4.1 Case studies

While the collection of pre-clinical *in vitro* data is necessary to further our understanding of BCC phage/bacterial dynamics, a crucial gap in knowledge regarding BCC phage therapy is human data, with only two compassionate use cases published (Aslam et al., 2019; Haidar et al., 2023) (Figure 2). Moreover, these published cases lack information on the phages used. BdPF16phi4281 was a phage that was produced specifically for the *B. dolosa* case (Aslam et al., 2019); however, no information regarding the phage’s genome, taxonomy, life cycle, or other factors was made available. Without this information, it is challenging to elucidate the phage characteristics essential for successful phage therapy, or possibly detrimental to treatment. In the *B. multivorans* case report, the genome of phage Bch7 was annotated, with no lysogeny or virulence genes described; however, limited genomic annotation of BCC phages, as detailed in this review, sheds doubt on the life cycle of *Bcepfunavirus* phages. This again underpins a need to enhance genomic characterisation of BCC phages to ensure safe phages for humans.

Encouragingly, in both case reports there were no detrimental immune responses or adverse events attributable to administration of phage therapy. In the *B. multivorans* case, phage administration resulted in a transient decrease in white blood cell count. However, this decrease was not correlated with any decrease in bacterial load in respiratory and blood cultures (Haidar et al., 2023). In the *B. dolosa* case, the patient tolerated both IV and nebulised phage formulations, with no adverse inflammatory responses after phage therapy (Aslam et al., 2019). These findings indicate that BCC phages are likely well tolerated and safe for human use and highlight the importance of monitoring human immune responses in phage therapy.

Another important consideration for phage therapy is the effect of human immune responses on bacteriophage efficacy. Phage inactivating antibodies from patients receiving phage therapy have been previously documented (Łusiak-Szelachowska et al., 2014), potentially decreasing phage concentration circulating in the body. In the, *B. multivorans* case, Bch7 load was quantified using quantitative real time PCR (q-PCR), detecting phage DNA from tracheal aspirate and BALF after 6 days. Combined with a lack of phage neutralising antibodies in patient sera (Haidar et al., 2023), these findings suggest that no immunological response could have impacted the efficacy or safety of phage therapy. In the *B. dolosa* case, this response was not monitored; however, viable phages were detected in patient sera and BALF starting on the 4th day of therapy, indicating a high titre of phages delivered to the site of infection (Aslam et al., 2019). This level of case reporting is essential for the treatment of future patients, as there is a lack of understanding of pharmacokinetics and pharmacodynamics of phages in the human body, especially for BCC phages.

Notably, both of these studies did not monitor the emergence of phage resistance (Aslam et al., 2019; Haidar et al., 2023). The emergence of resistance during infection may have impacted the outcomes of these cases, as both patients initially responded well to therapy before their infections worsened (Aslam et al., 2019; Haidar et al., 2023). In contrast, in a separate case of phage therapy used to treat *P. aeruginosa* lung infection that was detailed in one of these reports, phage resistance was monitored to adjust the therapy accordingly, adding a new phage to the treatment when the infection became resistant (Aslam et al., 2019). The patient eventually recovered and was discharged from hospital (Aslam et al., 2019). Measuring bacterial responses to phage treatment will be crucial to the future success of phage therapy and will allow for informed treatment modifications to counter phage resistance. However, due to limited availability of OL BCC phages, adjusting phage therapy in real time by switching therapeutic phage candidates may not be currently feasible. As an example, adjusting phage therapy for *B.dolosa* infection would be challenging, considering there are only 3 known phages with lytic activity against *B. dolosa* (Lynch et al., 2013; Lynch et al., 2010; Weiser et al., 2020).

In addition to adjusting phage therapy in real time, the timing of the initial administration of phage therapy may also affect efficacy of treatment. For example, in the *B. dolosa* case, there was a 3 month delay between initial screening of the clinical isolate and identification of a phage with lytic activity. This may have had an impact on the case outcome (Aslam et al., 2019), just as delayed antibiotic administration has been associated with increased mortality amongst individuals with pneumonia (Inchai et al., 2015; Heath et al., 1996). Ideally, shorter screening times could be facilitated by access to repositories of OL phages; however, as mentioned previously in this review, OL phages targeting BCC display narrow host ranges and appear to be exceedingly rare within existing reservoirs. This is where temperate BCC phages demonstrating bactericidal activity may be useful for patients requiring urgent treatment when OL phages are unavailable. This was performed in the *B. multivorans* case study, where a potentially temperate phage was safely administered within 10 days of initiation of phage screening (Haidar et al., 2023).

#### 6.4.2 Clinical trials

While data from compassionate use cases is relevant and important information for phage therapy, the validity of the data is limited by the lack of standardised case reporting. Ultimately, standardised clinical trials are essential for the complete translation of phage therapy as a whole, not just for BCC (McCallin et al., 2019) (Figure 2). Even before human trials, there needs to be a standardisation and consensus on how best to prepare and administer phage for clinical use. There are no standardised guidelines for phage manufacture (Pires et al., 2020); however, several criteria have been proposed (Pirnay et al., 2015). Firstly, preparation of phage formulations according to Good Manufacturing Practices (GMP) is essential to ensure safety of phage products (Pires et al., 2020). In addition, one of the most important considerations in producing safe phage formulations is the removal of bacterial toxins, such as LPS, bacterial debris, and bacterial DNA (Pires et al., 2020), as these can induce adverse side effects including sepsis (Cowdery et al., 1996). Periodic pH measurements, clarity tests and phage viability tests are also required to ensure long term safety of viability of produced phage throughout storage (Pires et al., 2020; Pirnay et al., 2015). Standardisation in these manufacturing processes should become the standard for preparing phage for use in treatment to ensure consistency in administration for lung infections.

Moreover, delivery of sufficiently high concentrations of phages into the lung microenvironment via intravenous administration is a challenge (Prazak et al., 2022). As a result, phage nebulisation has been explored as a potential delivery method for respiratory infections (Prazak et al., 2022). This appears to be a promising avenue for delivery of BCC phages as well, since these phages have been shown to be effective at decreasing bacterial load in the lungs of mice when delivered via a nose-only inhalation device (NOID). However, a concern with this approach is that phages are subject to degradation from the physical and chemical stresses during aerosolisation, including extreme temperature, mechanical stress, and changes in pH, which can compromise their bacterial killing capacity (Bodier-Montagutelli et al., 2017). Encouragingly, phage concentrations recovered from lung tissue of treated mice 2 days after either an intraperitoneal or NOID delivery of phage were similar, suggesting efficient delivery of phages to with both methods of administration (Semler et al., 2014). Other BCC phages have also demonstrated aerosolisation stability in *in vitro* simulated breathing models (Golshahi et al., 2008), and in dry powder formulations (Golshahi et al., 2011), indicating BCC phages have capacity to remain stable and effective when aerosolised. BCC phages should be evaluated for their stability in different formulations optimised for aerosolised delivery during treatment.

## 7 Conclusion

Phage therapy has emerged as one of the most promising alternatives to antibiotics to combat AMR in the post-antibiotic era. There remains limited data, and no established repositories of OL phages targeting BCC. Future work should seek to elucidate the mechanisms of phage infectivity in BCC, characterise lysogeny in BCC phages, explore methods to increase successful isolation of OL phages targeting BCC, and improve phage activity during treatment. Further assessment of the efficacy and safety of BCC phagess will provide crucial data and information on the feasibility of phage therapy to treat BCC infections in *CF.*
